# Multimorbidity and Functional Impairments in Chronic Life Stressors: Evidence From the Health and Retirement Study

**DOI:** 10.1093/geroni/igaf122.2029

**Published:** 2025-12-31

**Authors:** Ayse Malatyali, Tom Cidav, Abigail Tice, Rui Xie, Abbie Ashton, Michael Dino, Lisa Wiese

**Affiliations:** University of Central Florida, Orlando, Florida, United States; University of Central Florida, Orlando, Florida, United States; University of Central Florida, Orlando, Florida, United States; University of Central Florida, Orlando, Florida, United States; University of Central Florida, Orlando, Florida, United States; University of Central Florida, University of Central Florida, Florida, United States; Florida Atlantic University, Boca Raton, Florida, United States

## Abstract

Chronic Life Stressors (CLS) significantly impact the well-being of older adults, yet their health-related determinants remain underexplored. This study examines associations between multimorbidity, functional impairments, and CLS exposure. Using logistic regression models, we analyzed cross-sectional data from 2,822 older adults in the 2020 Health and Retirement Study. CLS was assessed across seven domains: physical-emotional stress, substance use in the family, work-related stress, financial strain, housing instability, strained relationships, and caregiving burden and was categorized as no exposure, moderate exposure (1–2 stressors), and severe exposure (3+ stressors). Multimorbidity was defined as having up to seven chronic conditions. Functional impairments included difficulties with Instrumental Activities of Daily Living (IADL) and mobility limitations. Older adults ≥75 years had lower odds of severe CLS than those aged 65–74. Women had a significantly higher risk of severe CLS exposure (OR = 1.20) than men. African American individuals had lower odds of moderate CLS (OR = 0.61) but no significant difference in severe CLS. Hispanic ethnicity was not significantly associated with CLS. Multimorbidity significantly predicted CLS; Individuals having three or more chronic conditions were 2.68 times more likely to report severe CLS exposure, and those with two conditions were 2.55 times more likely to report severe CLS exposure. Individuals with IADL difficulties had a 2.34 times increased risk of severe CLS, while those with mobility limitations had 2.07 times higher odds of severe CLS exposure. These findings underscore the need for targeted interventions to address multimorbidity and functional limitations, reducing the burden of CLS in aging populations.

